# Supplementary material for: Superhard bulk high-entropy carbides with enhanced toughness via metastable in-situ particles
Source: Nat Commun. 2023 Sep 15;14:5717. doi: 10.1038/s41467-023-41481-6 (PMC10504279; doi:10.1038/s41467-023-41481-6)
Supplement: Supplementary file 1 — Supplementary Information [file 41467_2023_41481_MOESM1_ESM.pdf]

Supplementary Information

Superhard bulk high-entropy carbides with enhanced toughness via metastable in-situ particles

Jiaojiao Hu, Qiankun Yang, Shuya Zhu, Yong Zhang, Dingshun Yan, Kefu Gan, Zhiming Li*

School of Materials Science and Engineering, Central South University, Changsha, China

*Correspondence to: lizhiming@csu.edu.cn

This file includes:

Supplementary Fig. 1 to 6

Supplementary Table 1 to 2

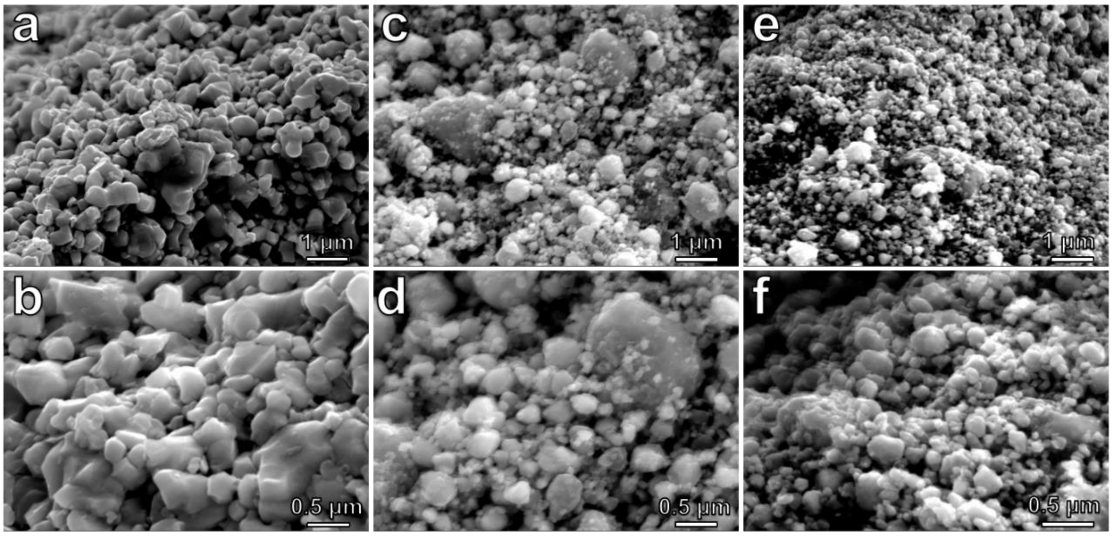

Supplementary Fig. 1 SEM images of mixed powders with different ball-milling times.

a-b 0 h; **c-d** 20 h; **e-f** 110 h.

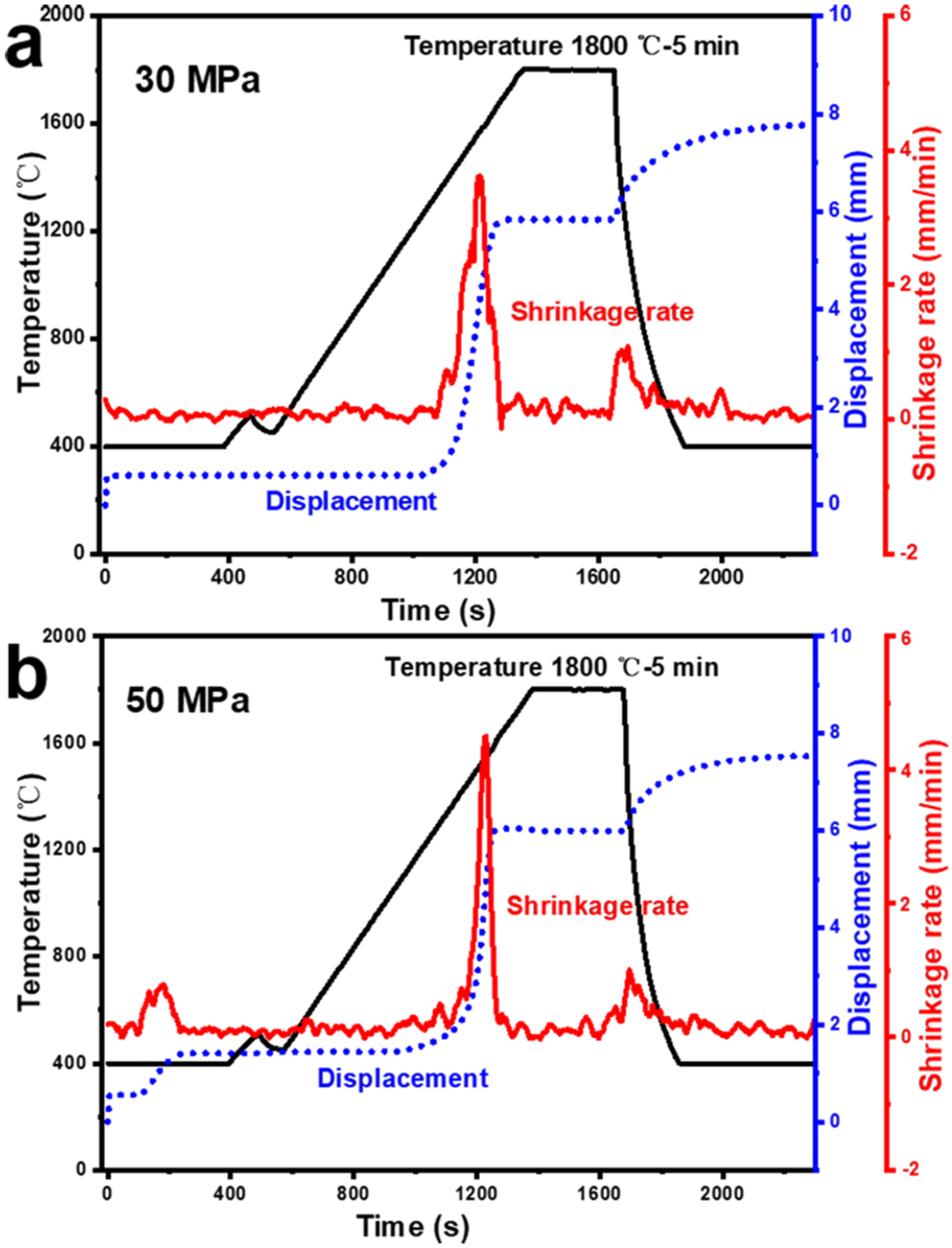

Supplementary Fig. 2 The recorded sintering curves for the (WTaNbZrTi)C HEC samples. a HEC sintered at 30 MPa. **b** HEC sintered at 50 MPa.

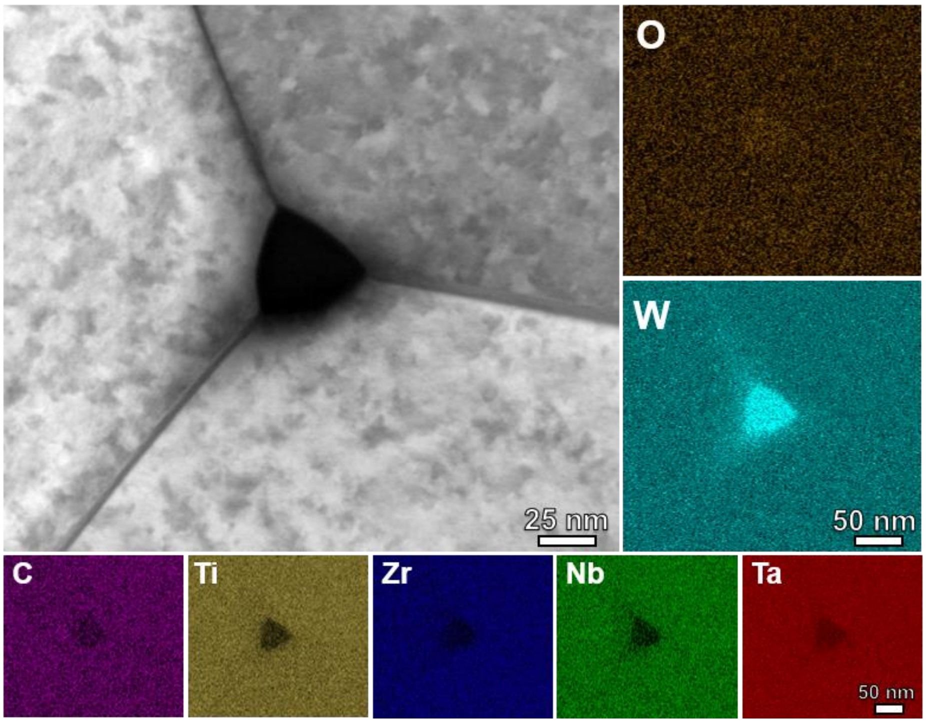

Supplementary Fig. 3 BF STEM image of a grain boundary triple junction, and the corresponding EDS mappings.

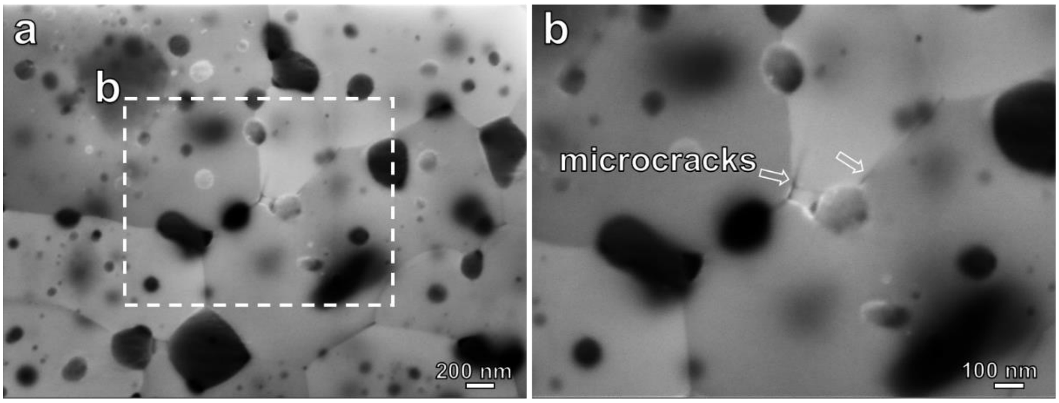

Supplementary Fig. 4 The pre-existing microcracks in the vicinity of certain ZrO_2 particles in as-sintered sample. **a** SEM image at low magnification of HEC sintered at 30 MPa. **b** Zoom-in image of the white dashed square region in a.

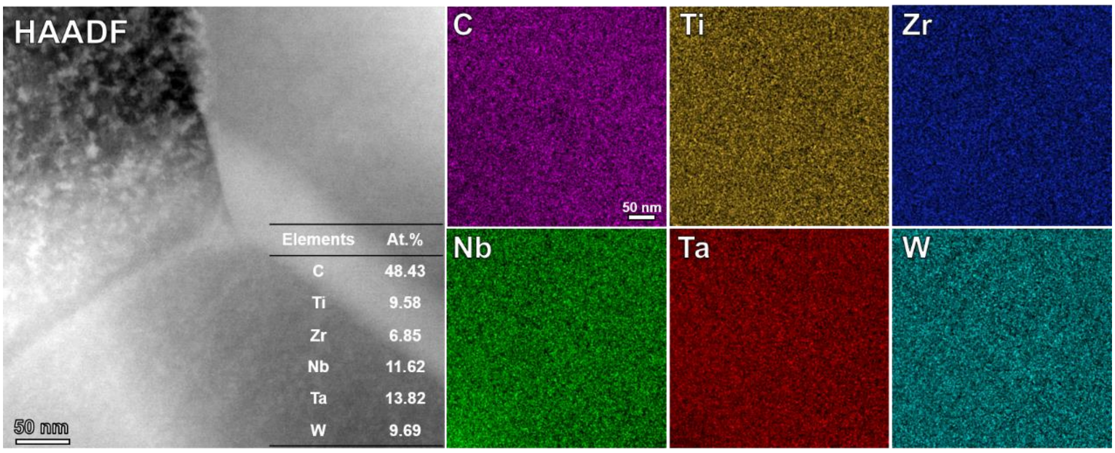

Supplementary Fig. 5 HAADF-STEM image of a sample region in the (WTaNbZrTi)C HEC sintered at 30 MPa, and the corresponding EDS mappings. The quantitative results from the compositional analysis for the HEC matrix are shown on the STEM image.

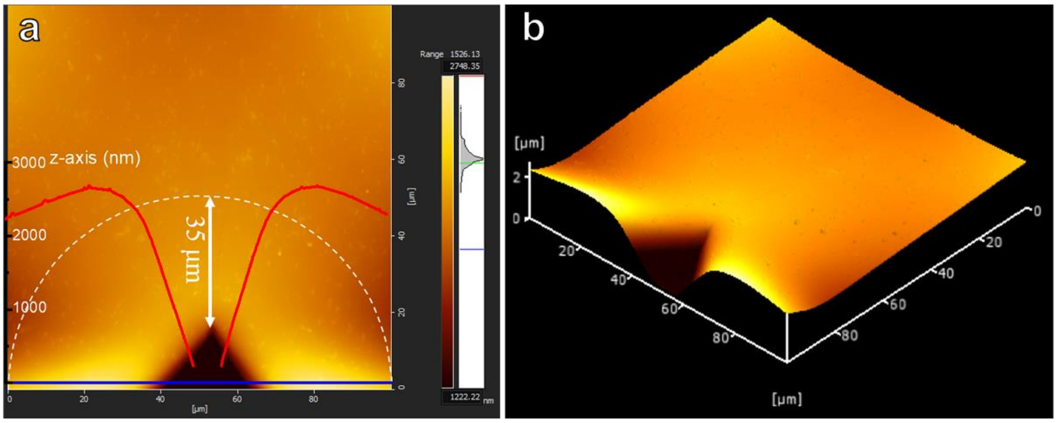

Supplementary Fig. 6 Atomic force microscopy (AFM) images showing the transformation zone developed about a Vickers indentation with a load of 30 kg. a 2D AFM image, and the insert red profile line corresponds to the position of a line in blue. The transformation zone diameter can be estimated from the white dash semicircle. **b** Corresponding 3D profile.

Supplementary Table 1 Vickers hardness and fracture toughness of individual monocarbides and various HECs. The values of HECs reported previously and that calculated from the rule of mixture (ROM) are shown for comparison. The specific loads and methods for measuring hardness and fracture toughness are provided in the brackets following the values. The details of references are shown in the reference list of the manuscript.

Samples	Hardness (GPa)	K_{IC} ($MPa \cdot m^{1/2}$)	References
**WC**	24.2 (98.1 N)	4.40 ± 0.1 (98.1 N)	[37]
**TaC**	13.9 (9.8 N)	2.7 ± 0.3 (9.8 N)	[38]
**NbC**	19.7 ± 1.9 (9.8 N)	2.9 ± 0.2 (98 N)	[39]
**ZrC**	17.6 ± 0.14 (9.8 N)	3.3 ± 0.03 (19.6 N)	[40]
**TiC**	21.87 ± 1.02 (49 N)	3.71 ± 0.22 (49 N)	[41]
**ROM**	19.5	3.4	
**(WTaNbZrTi)C-50 MPa**	21.0 ± 0.1 (9.8 N)	5.4 ± 0.2 (49 N)<br>5.89 ± 0.19 (SENB method)	This work
**(TiZrNbTaMo)C**	25.3 (9.8 N)	3.3 (98 N)	[43]
**(ZrNbTiV)C**	20.8 (9.8 N)	4.7 ± 0.5 (49 N)	[19]
**(TiZrNbTaHf)C**	18.8 (9.8 N)	3.0 (9.8 N)	[33]
**(VNbTaMoW)C**	21.2 (9.8 N)	3.6 (SENB method)	[42]
**(NbTaMoW)C**	21.2 (9.8 N)	3.6 (SENB method)	[44]
**(TiNbTaHfV)C**	21.5 (9.8 N)	4.8 (SENB method)	[24]

Supplementary Table 2 Structural parameters of the various reported HECs. The parameters including valence electron concentration (VEC), entropy forming ability (EFA) and atomic-size difference (δ_a), and the experimentally observed phase structures. The details of references are shown in the references list of the manuscript.

HEC	VEC (f.u.)	EFA[15] $(eV/atom)^{-1}$	δ_a (%)	Single or Multiple Phase
**(HfNbTaTiZr)C**	8.4	100	2.96	Single[24, 7, 16]
**(HfNbTaTiV)C**	8.6	100	3.72	Single[24, 16]
**(HfTaTiWZr)C**	8.6	50	4.89	Single[16]
**(TiZrHfTaMo)C**	8.6	63	4.94	Single[16]
**(TiHfNbTaMo)C**	8.8	83	4.14	Single[16]
**(TiZrNbTaMo)C**	8.8	71	4.53	Single[16]
**(HfMoTiWZr)C**	8.8	37	5.90	Multiple[16]
**(HfNbTaTiW)C**	8.8	67	4.08	Single[16]
**(NbTaTiVW)C**	9.0	77	3.63	Single[16]
**(HfMoTaWZr)C**	9.0	45	5.88	Multiple[16]
**(HfMoVWZr)C**	9.0	38	6.30	Multiple[16]
**(TiVNbTaMo)C**	9.0	100	3.67	Single[16]
**(MoNbTaVW)C**	9.4	125	4.13	Single[16, 42]
**(WTaNbZrTi)C**	8.8	59	4.48	/
